# Short-term use of ceftriaxone sodium leads to intestinal barrier disruption and ultrastructural changes of kidney in SD rats

**DOI:** 10.1080/0886022X.2023.2230322

**Published:** 2023-07-19

**Authors:** Wenli Zou, Yueming Liu, Wei Zhang, Bo Lin, Wei Shen, Yiwen Li, Qiang He, Juan Jin

**Affiliations:** Urology & Nephrology Center, Department of Nephrology, Zhejiang Provincial People’s Hospital, Affiliated People’s Hospital, Hangzhou Medical College, Hangzhou, Zhejiang, China

**Keywords:** Ceftriaxone sodium, gut microbiota, intestinal barrier, transmission electron microscopy

## Abstract

**Objective:**

Antibiotic treatments are known to disturb gut microbiota, but their effects on the mucosal barrier and extraintestinal diseases are rarely discussed. The aim of this study was to evaluate and visualize the impact of antibiotics on colonic mucus and the microbial community, and to assess whether intestinal dysbacteriosis is involved in the pathogenesis and progression of extraintestinal diseases *in vivo*.

**Materials and Methods:**

Twenty-one SD rats were randomly assigned into three groups followed by different experimental treatments. The albumin–creatinine ratio, urinary protein and occult blood semi-quantified test were tested. Fecal samples were collected at different time points (0,4, and 12 weeks) for 16S rRNA gene sequencing. Colon and kidney specimens were examined using light microscopy and transmission electron microscopy (TEM) to identify morphological changes.

**Results:**

Ceftriaxone intervention for one week did not cause any symptoms of diarrhea or weight loss, but the alpha and beta diversities of gut microbiota decreased quickly and significantly, a lower Firmicutes/Bacteroidetes (F/B) ratio was observed. At week 12, although the alpha and beta diversities increased to a level similar to that of the control (CON) group, LEfSe analysis indicated that the microbial community composition still differed significantly in each group. In addition, KEGG metabolic prediction revealed different metabolic functions in each group. TEM examination of colon revealed that dramatic morphological changes were observed in the ceftriaxone (Cef) group, wherein microvilli were misaligned and shortened significantly and morphologically intact bacteria were seen on the epithelial cell surface. TEM examination of kidneys from the Cef group showed characteristic glomerular changes in the form of widely irregularly thickened glomerular basement membrane (GBM) and foot process fusion or effacement; mild thickening of the GBM and foot process fusion was detected when ceftriaxone and Resatorvid (TAK242, an inhibitor of TLR4 signaling) are used together in the ceftriaxone + TAK242 (TAK) group.

**Conclusions:**

Short-term use of ceftriaxone induced dynamic changes of gut microbiota and lead to intestinal barrier disruption and ultrastructural changes of kidneys in the SD rats. Moreover, interference with the TLR4-dependent signaling pathway can alleviate the damage to the intestinal barrier and kidney.

## Introduction

The gastrointestinal tract is colonized by a dense community of commensal microorganisms, referred to as the intestinal microbiota, that plays an important role in the physiological processes related to immunity and metabolism. Widespread antibiotic administration is probably a major factor contributing to rapid changes in the gut microbiota. Both short- and long-term use of ceftriaxone sodium has been found to induce microbiota dysbiosis and immune homeostasis disorders [1,2]. Dysbiosis may cause the development of various gastrointestinal diseases, including inflammatory bowel disease, colon cancer, and other intestinal diseases such as obesity and chronic kidney disease (CKD) [3]. Emerging evidence suggests that intestinal dysbiosis–associated gut barrier disruption and aberrant mucosal immunity are important for systemic inflammation and are risk factors for CKD, although the mechanisms underlying its pathogenesis remain unclear [4–6]. Related to this, TLR4 is an important toll-like receptor (TLR) that recognizes pathogen-associated molecular patterns, especially from gram-negative bacteria, and controls intestinal epithelial cells and the mucosal barrier [7,8]. The current study was conducted to assess the effect of short-term use of ceftriaxone sodium on the intestinal microbiota and explore whether antibiotic use–induced changes in the intestinal microbiota lead to intestinal barrier disruption and ultrastructural changes in the kidneys of SD rats. We also believe that interfering with the TLR4-dependent signaling pathway will alleviate damage to the intestinal barrier and kidney.

## Materials and methods

### Animals and treatments

Twenty-one weaning SPF-grade male Sprague Dawley (SD) rats were obtained (average weight: 113.1 g). The SD rats were kept and fed in a clean room at a constant temperature (18 °C) and humidity (45%) with a 12-h light/dark cycle. Routine urine testing was performed after one week of pre-feeding using the test strip method. Rats that showed no hematuria or proteinuria were selected after three days of adaptation. All animal experiments were performed under institutionally approved protocols by the Institutional Animal Care and Use Committee, ZJCLA (approval number: ZJCLA-IACUC-20020021).

The SD rats were randomly assigned into three groups of seven, namely, the control (CON), ceftriaxone (Cef), and ceftriaxone + TAK242 (TAK) groups. All were administered bovine serum albumin (BSA) and carbon tetrachloride (CCl_4_) by gavage according to the IgAN modeling methods [9–11], Briefly, the immunogen BSA was intragastrically administered at a dose of 400 mg/kg once every other day for eight consecutive weeks, and 0.1 mL of CCl_4_ with 0.3 mL of castor oil was administered once weekly for eight weeks. According to literature reports about IgAN modeling, the immunogen BSA and CCl4 were administered to increase intestinal immune response and reduce the ability of the liver to clear intestinal toxin, respectively. In the Cef and TAK groups, ceftriaxone was administered orally at a dosage of 8 g/kg once a day for a week at the beginning, and the rats in the other group were administered sterile water intragastrically as controls [1,12]. TAK242 dissolved in 20% lipovenoes at 3 mg·kg^−2^d^−1^ (7.5 mL/kg) was injected into rats in the TAK group for a week at the beginning, and the rats in the other groups received intravenous injection of the same amount of lipovenoes by tail veins [13,14]. Non-intravenous administration of ceftriaxone and lipovenoes was done to reduce direct damage to the kidney.

### Sample collection and preparation

At the end of the study, 4-h urine samples were collected using metabolic cages. Kidney samples and the initial segment of the colon were obtained and subjected to light microscopy and transmission electron microscopy (TEM). Rat fecal samples were collected at different periods of the experiment (0,4, and 12 weeks) and frozen at −80°Cuntil DNA extraction and subsequent 16S rRNA gene sequencing.

### Measurement of body weight, liver, and kidney index

The animals were weighed prior to weekly feeding, and the weight change trends of the three groups were monitored. The liver and kidneys were harvested and measured, and the respective indices were calculated as follows: liver index (%) = [liver weight (g)/body weight (g)] × 100% and kidney index (%) = [kidney weight (g)/body weight (g)] × 100%.

Urine analysis. At week 12, 4-h urine samples were collected in metabolic cages to estimate the urine index. Micro protein PRM kit (CS7480; Leadmanbio, Beijing, China) was used for the measurement of urinary micro protein. The urinary protein reacts with pyrogallol red-molybdate dye reagent to form blue purple colored complex with maximum absorbance at 600 nm. The assay procedure was performed as described by the manufacturer. Dry chemistry test strips (URIT 8 V; Guilin, China) were used to semi-quantitatively detect hematuria and urinary protein. The test procedure was performed as described by the manufacturer. The color was read exactly after 2 min. Colors formed range from yellow for a negative reaction to yellow green and green to blue green for a positive reaction both for the hematuria and urinary protein.

### Illumina MiSeq sequencing

Rat feces samples (50–200 mg) were collected in a germ-free environment and frozen at −80 °C until DNA extraction. Total DNA was extracted from the fecal samples, genomic DNA integrity and quality were detected and measured using agarose gel electrophoresis and NanoDrop 2000, respectively. The amplification primers used were according to the target regions and sequencing adaptors were attached to the ends of the primers for polymerase chain reaction (PCR) amplification. The PCR products were purified, quantified, and homogenized to form a sequencing library that was subsequently sequenced using the MiSeq platform and 2 × 250 bp double-ended sequencing strategy, followed by bioinformatics analysis. To obtain high-quality sequencing data and improve the accuracy of subsequent bioinformatics analysis, the DADA2 plug-in of QIIME2 software was used to filter, denoise, merge, and de-chimer data.

### Colonic and kidney ultrastructures

The colons and kidneys of rats were collected after euthanization, and one part of each sample was fixed in 4% paraformaldehyde, embedded in paraffin, cut into 4-mm sections, and stained with hematoxylin and eosin (HE) for light microscopy. The remaining parts of the samples were subjected to TEM analysis as follows: samples were fixed in 2.5% glutaraldehyde in Hanks’ balanced salt solution (pH = 7.0) at 4 °C for 2 h. Subsequently, tissues were post-fixed in 1% OsO_4_ in Hanks’ solution at 4 °C for 2 h, block-stained in 2% uranyl acetate solution at 40 °C for 1 h, dehydrated in ethanol and acetone, and embedded in Spurr’s resin. Ultrathin sections were obtained using an ultramicrotome (LKB-8800; LKB, Bromma, Sweden) and were stained with uranyl acetate and lead citrate. The JEM-100C microscope (JEOL, Tokyo, Japan) was used to examine the sections. All tissue sections were examined by two pathologists.

### Statistical analysis

All values are expressed as the mean ± standard error, and statistical analyses were performed using SPSS version 22.0 (IBM Corp.). Values were compared among the three groups using analysis of variance (ANOVA), and a *post hoc* least significant difference test was performed when ANOVA indicated significance. If the indices were ordered categorical variables, the values were compared using the Wilcoxon rank sum test. *p* < 0.05 was considered statistically significant.

## Results

### General conditions of the animals

**Figure 1.**
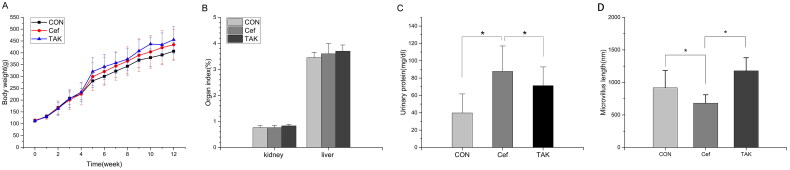
Indicators change over 12 week in rats. (A)Weight, (B) liver and kidney index. (C) Urine indicators(mg/dl). (D) Microvillus length(nm).

During the experiment, all the rats in the three groups were in good condition and had no diarrhea. The overall weight of the three groups of rats showed an increasing trend (Figure 1A). Compared with the CON group, the body weights of the Cef and TAK groups were not significantly different. Liver weight/body weight and kidney weight/body weight ratios showed no significant differences among the three groups (Figure 1B). After ceftriaxone intervention for just one week, the urinary protein and occult blood semi-quantified tests showed no significant differences, but urinary protein in the Cef group was significantly increased compared to that in the CON group (Figure 1C).

### Intestinal bacterial structure and relative abundance

**Figure 2.**
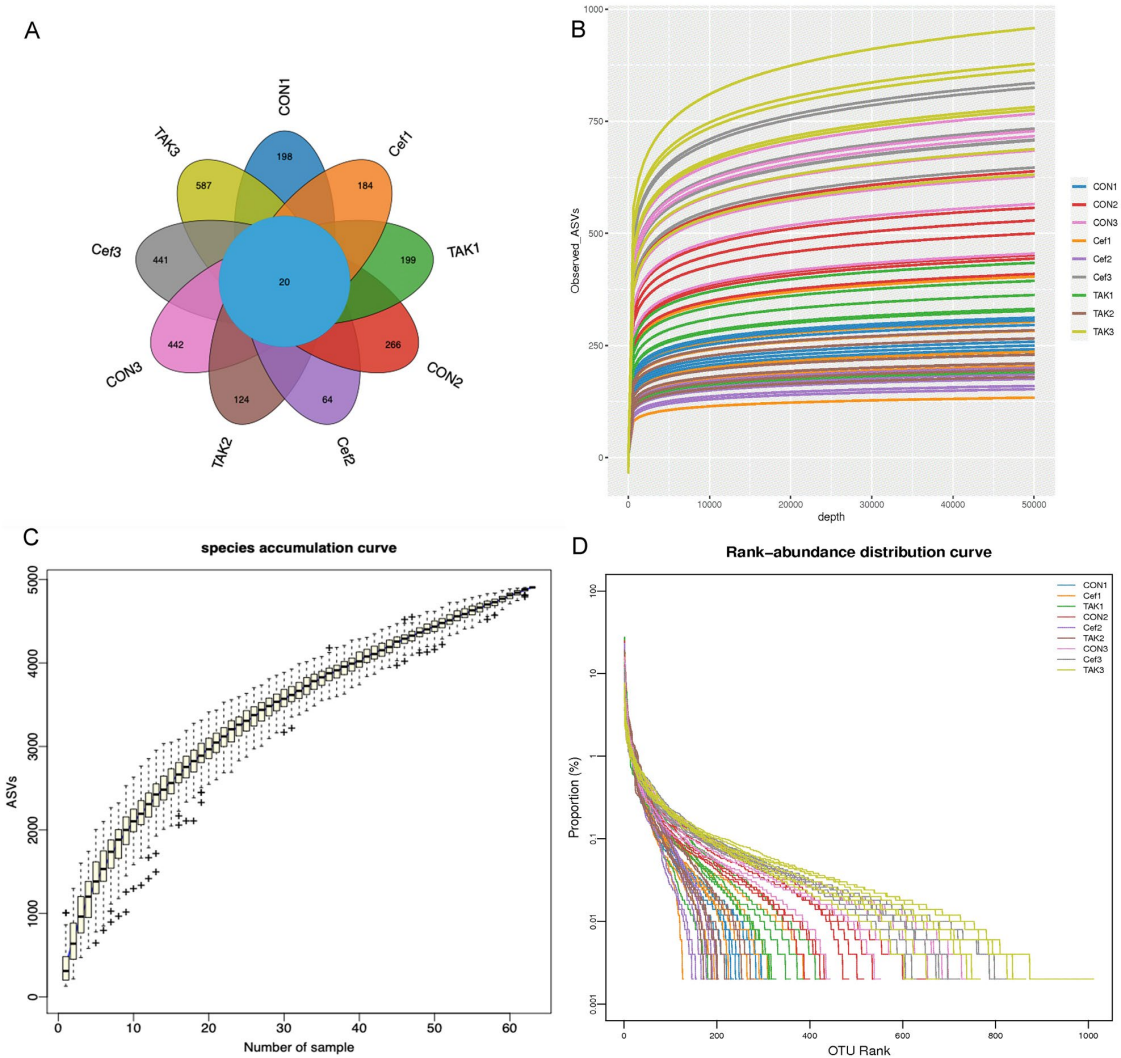
OUT Division of fecal sample microecology. (A)Venn diagram, (B) Rarefaction curves, (C) Species accumulation curves, (D) Rank-abundance distribution curves.

We performed sequencing of 21 samples using the Illumina MiSeq platform at three time points (0, 4, and 12 weeks). The Venn graph (Figure 2A) showed that the three groups had a similar level of OTUs (operational taxonomic units) at week 0, among which there were 198 in the CON group, 184 in the Cef group, and 199 in the TAK group. At week 4, the CON group increased significantly compared with the Cef and TAK groups. At week 12, all groups increased to a similar level of OTUs. The rarefaction, species accumulation curves, and abundance registration curves tended to be stable, which indicated that the current results are sufficient to reflect the diversity of the current sample and the average degree to be sufficiently high (Figure 2B–D).

**Figure 3.**
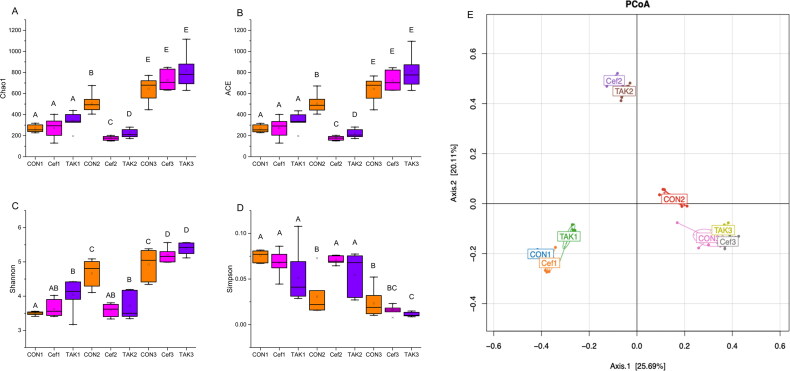
Alpha-diversity analysis and beta diversity of the fecal sample. (A) Chao 1, (B) ACE, (C) Shannon, (D) Simpson (E) PCoA analysis.

In terms of alpha diversity (Figure 3A–D), the microbiome of the CON group showed increased richness and diversity over a 12-week period; however, the Cef and TAK groups, after one week of ceftriaxone administration, showed significantly decreased richness and diversity at week 4, but after feeding for 12 weeks, the richness and diversity showed no differences compared with the CON group. The beta diversity of the gut microbiota in the three groups was measured by principal coordinate analysis, which showed the degree of similarity among the three microbial communities. The results revealed that at 0 and 12 weeks, the distribution structures of the three groups in space were similar. At 4 weeks, the distribution structure of the Cef and TAK groups showed distinct differences in clustering with the CON group (Figure 3E).

**Figure 4.**
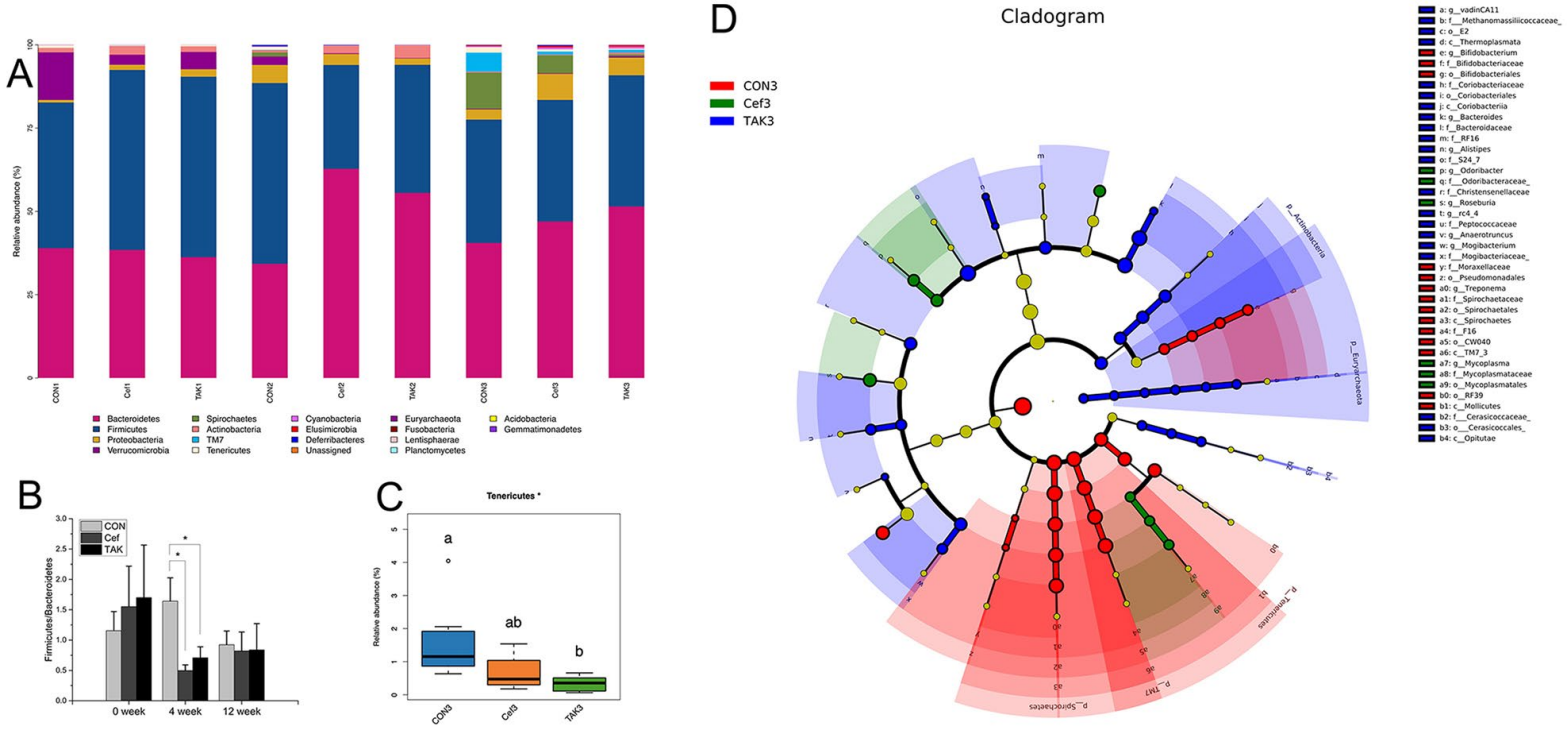
Analysis the characteristic of the flora at the phylum and genus level. (A) The structure and relative abundance of the flora at the phylum, (B) Firmicutes/Bacteroidetes (F/B) ratio, (C) relative abundance of Tenericutes at the time of week 12, (D) LEfSe analysis.

The structure and relative abundance of the flora are shown at the phylum level (Figure 4A), and the major bacterial phyla were Bacteroidetes, Firmicutes, Proteobacteria, Verrucomicrobia, and Spirochetes. Compared with the CON group, the Firmicutes/Bacteroidetes (F/B) ratio in Cef and TAK groups decreased significantly at the fourth week, although there was no significant difference at weeks 0 and 12 among the three groups (Figure 4B). At week 4, compared with the CON group, the abundance of Bacteroidetes in both the Cef and TAK groups increased significantly, as with Actinobacteria the TAK group, but not in the Cef group. The phyla Firmicutes, Verrucomicrobia, Spirochetes, TM7, Tenericutes, and Elusimicrobia were decreased significantly in both the Cef and TAK groups. Phyla Proteobacteria and Deferribacteres were significantly decreased in the TAK group, but not in the Cef group. Phyla Cyanobacteria was significantly decreased in the Cef group, but not in the TAK group. At week 12, the relative abundance of the above phyla were at a similar level among the three groups, with only Tenericutes showing significant differences between the CON and TAK groups (Figure 4C). LEfSe analysis (Figure 4D) was performed by submitting a relative abundance matrix at the genus level and showed the enriched bacteria for each group at the following taxonomic levels: class, order, family, and genus. At the family level, Methanomassiliicoccaceae, Coriobacteriaceae, Bacteroidaceae, RF16, S24-7, Christensenellaceae, Peptococcaceae, Mogibacteriaceae, and Cerasicoccaceae were more prevalent in the TAK group; Bifidobacteriaceae, Moraxellaceae, Spirochaetaceae, and F16 were more prevalent in the CON group; and Odoribacteraceae and Mycoplasmataceae were more prevalent in the Cef group. At the genus level, vadinCA11, *Bacteroides*, *Alistipes*, rc4_4, *Anaerotruncus*, and *Mogibacterium* were more prevalent in the TAK group; *Bifidobacterium* and *Treponema* were more prevalent in the CON group; and *Odoribacter*, *Roseburia*, and *Mycoplasma* were more prevalent in the Cef group.

### Intestinal flora function

**Figure 5.**
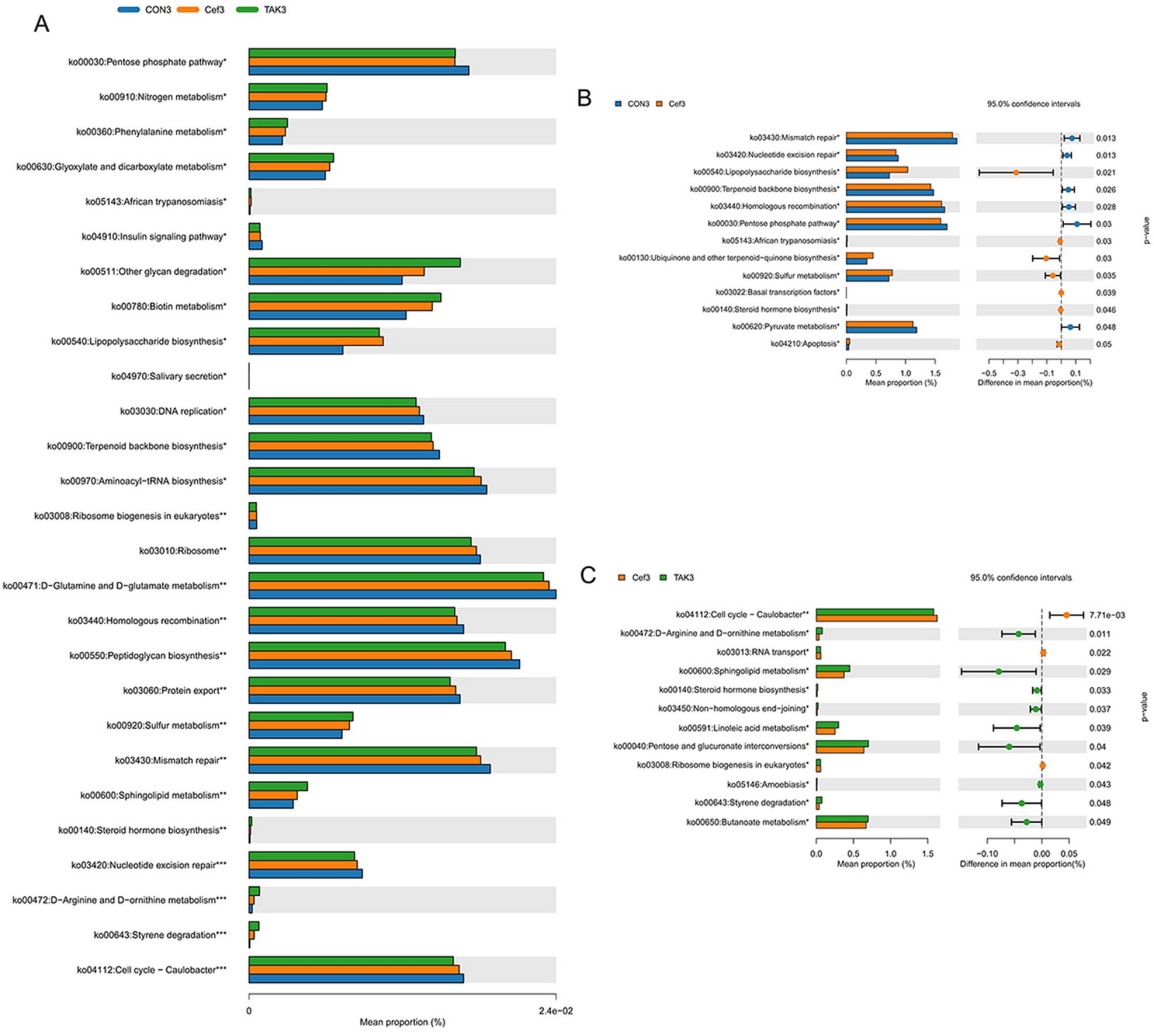
Prediction of metabolic function of intestinal flora. (A) Prediction second level distribution diagram of KEGG by PICRUSt2, (B) Predicted functional comparison between CON group and Cef group at week 12, (C) Predicted functional comparison between TAK group and Cef group at week 12.

Based on the full-length sequence of the 16S rDNA gene of the tested microbial genome, the Greengenes 16S rRNA gene full-length sequence database was used to predict their metabolic function. KEGG metabolic prediction showed that the higher average relative abundance of each function in each sample group was D-Glutamine and D-glutamate metabolism, peptidoglycan biosynthesis, and mismatch repair (Figure 5A). Compared with the CON group at week 12, the flora of the Cef group showed significantly higher metabolic function in lipopolysaccharide biosynthesis, African trypanosomiasis, ubiquinone and other terpenoid-quinone biosynthesis, sulfur metabolism, basal transcription factor, steroid hormone biosynthesis, and apoptosis, and showed significantly lower metabolic function in mismatch repair, nucleotide excision repair, terpenoid backbone biosynthesis, homologous recombination, pentose phosphate pathway, and pyruvate metabolism (Figure 5B). When compared with the Cef group at week 12, the flora of the TAK group showed significantly higher metabolic function in D-arginine and D-ornithine metabolism, sphingolipid metabolism, steroid hormone biosynthesis, non-homologous end-joining, linoleic acid metabolism, pentose and glucuronate interconversions, amebiasis, styrene degradation, and butanoate metabolism, and significantly lower metabolic function in the cell cycle–*Caulobacter*, RNA transport, and ribosome biogenesis in eukaryotes (Figure 5C).

### Colonic ultrastructures

**Figure 6.**
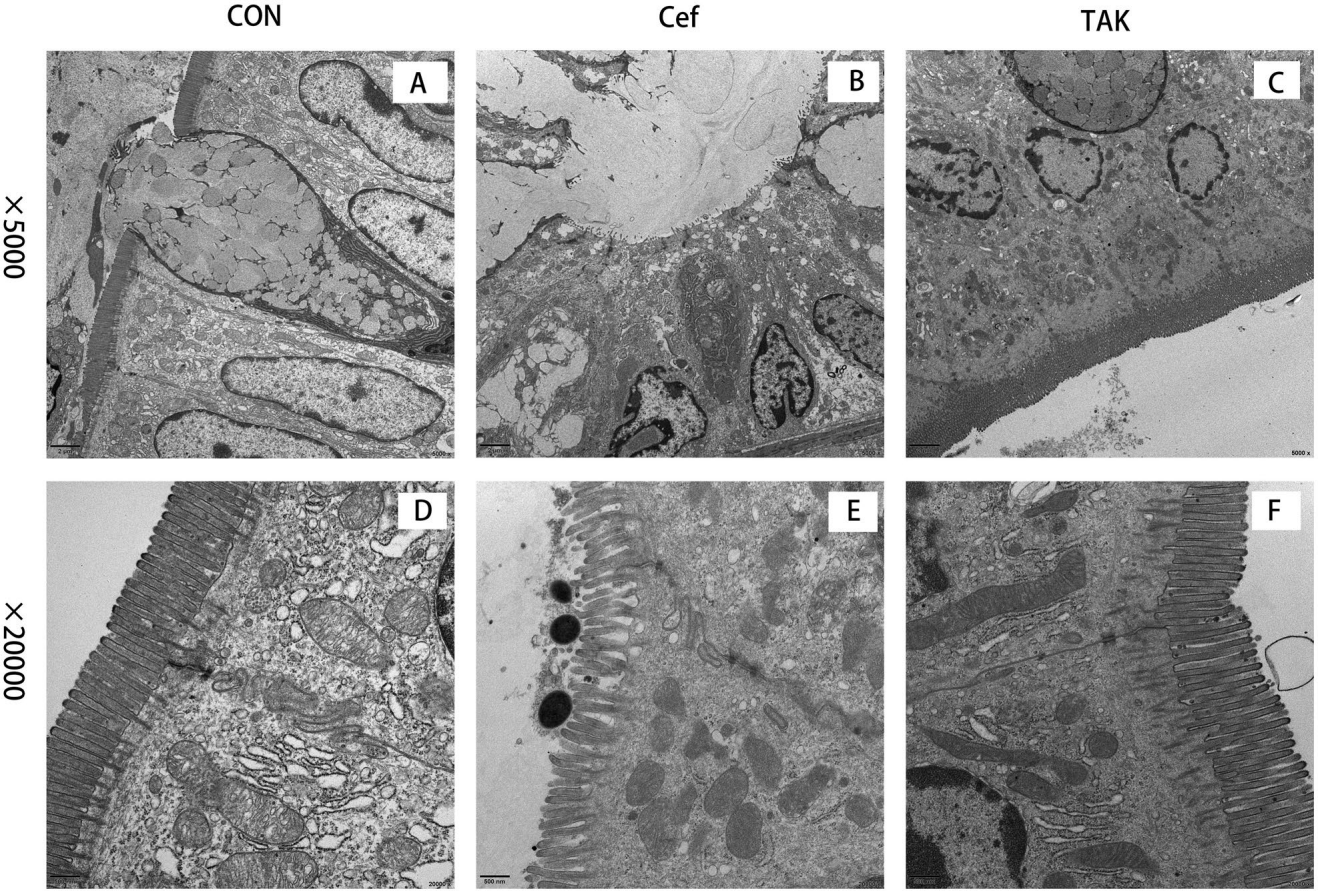
Intestinal mucosal morphological changes. (A-C)colon section of the CON, Cef, and TAK at low magnification, (D-F) Colon section of the CON, Cef, and TAK at high magnification.

To evaluate intestinal barrier function, we examined the initial segment of the colon mucosa using TEM and measured intestinal villus length. At high magnification, tight junctions between epithelial cells showed normal morphology in all the colon sections in all groups. the rats of the CON and TAK groups, the surface microvilli of the mucosal epithelium of rat colon were uniform in size and showed a neat arrangement (Figure 6A and C). Dramatic morphological changes in the colon were observed in rats in the Cef group, where the microvilli were misaligned, and morphologically intact bacteria were observed on the epithelial cell surface (Figure 6B). These results indicate that epithelial junction integrity was not compromised after short-term antibiotic treatment. Compared with the CON and TAK groups, the villus in the CEF group was significantly shortened (Figure 1D).

### Kidney ultrastructures

**Figure 7.**
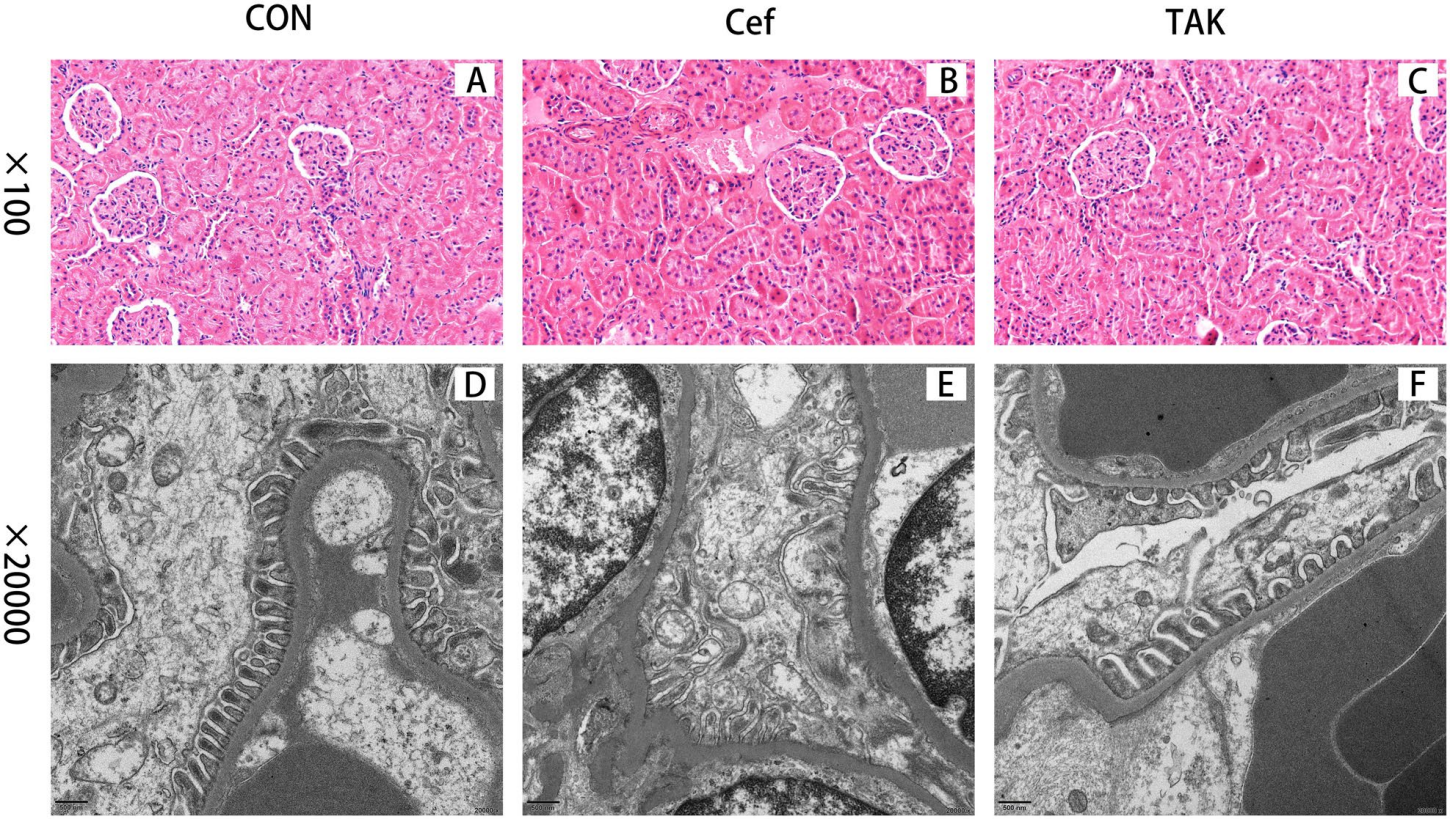
Representative micrographs for Histopathological changes of kidney from the three groups. (A-C) Representative features of CON, Cef, TAK group with light microscopy, (D-F) electron micrographs.

Light microscopic examination of HE-stained kidney sections from the three groups showed normal histological architecture of both glomeruli and renal tubules (Figure 7A–C).TEM examination of control kidneys showed glomeruli that appeared as glomerular capillaries with approximately intact glomerular basement membrane (GBM) and epithelial cells (podocytes) with approximately normally distributed foot processes, although focal fusions of foot processes of podocytes were occasionally observed (Figure 7D). In the Cef group, characteristic glomerular changes were in the form of widely irregularly thickened GBM and foot process fusion or effacement, and cytoplasmic vacuoles were more prominent (Figure 7E). Mild thickening of the GBM and foot process fusion was also detected in the TAK group (Figure 7F).

## Discussion

Antibiotics are effective in treating bacterial infections; however, they also act on commensal bacteria and can result in the alteration of gut microbial composition, leading to dysbiosis. Emerging evidence suggests that intestinal dysbiosis is associated with diverse pathological processes [15–17]. Ceftriaxone sodium, a third-generation cephalosporin, has broad-spectrum activity against gram-positive and gram-negative bacteria. In our study, after short-term use of ceftriaxone sodium (7 days), weight gain, food, and water intake, and shape of feces showed no differences between control and antibiotic-treated mice.

It has been reported that antibiotic use can lead to short-term or long-term effects on gut microbiota, although little is known about these [18,19]. Our data showed dynamic changes in the intestinal microbiota after ceftriaxone use based on high-throughput sequencing. In our study, antibiotic use decreased the diversity and abundance of intestinal bacteria immediately. At week 4, almost all the bacteria at the phylum level showed a downward trend, except for Bacteroidetes. Firmicutes and Bacteroidetes were the most abundant phyla in the gut microbiota. Various animal and human studies have revealed an increase in Firmicutes and a decrease in Bacteroidetes concentrations (thus an increase in F/B ratio) in obese subjects [20,21]. It has also been reported that an increase in Bacteroidetes and decrease in Firmicutes are markers of dysbiosis in patients with Crohn’s disease [22,23]. Our data showed a lower F/B ratio in the antibiotic treatment groups, which is in accordance with the literature, and a lower F/B ratio can be considered as a potential marker of dysbiosis. Although the abundance and diversity of bacteria successfully recovered to the same level as the control at 12 weeks, only the Tenericutes remained at a lower level in the TAK groups. LEfSe analysis indicated that the microbial community composition still differed in each group when analyzed at the levels of class, order, family, and genus. In addition, KEGG metabolic prediction revealed different metabolic functions in each group. Short-term use of ceftriaxone sodium was found to induce microbiota dysbiosis and metabolic function differences after a longer period of antibiotic drug discontinuation.

Given that antibiotics can alter the gut microbial composition and cause intestinal barrier dysfunction, we were interested in investigating the colonic ultrastructural changes elicited by its short-term use. Compared to previous light microscopy–based findings, our approach using TEM provided detailed results with a higher resolution. The rats in the Cef group still had misaligned and shortened microvilli after 11 weeks of antibiotic discontinuation, which suggests that short-term antibiotic use caused long-term damage to the intestinal barrier. Goblet cells are responsible for the secretion of mucin, providing mucosal surfaces with a thick mucus lining, which acts as a barrier to limit interactions with liminal microbes and, in response to bacterial components, most can trigger the rapid release of mucin and quickly restore the mucus barrier [24,25]. Mucin production has been reported to be affected by microbial changes, and the genera *Odoribacter*, *Roseburia*, and *Mycoplasma*, which were enriched in the Cef group, may be an important factor affecting mucus production. There has been little literature about the effect of these three bacterial genera on the intestinal barrier, and more research is needed to study the relationship between intestinal flora and the intestinal mucosal barrier.

Interestingly, the rats in the TAK group, which also received antibiotics, showed less change in colonic ultrastructure than that in the Cef group. Recent evidence shows that TLRs recognize specific patterns of microbial components, especially those from pathogens, and regulate the activation of both innate and adaptive immunity, and participate in maintaining colonic homeostasis [26,27]. Among these TLRs, TLR4 recognizes lipopolysaccharide (LPS) in particular and is primarily involved in the control of gram-negative bacteria. Resatorvid (TAK-242), an inhibitor of TLR4 signaling, inhibits the production of LPS-induced inflammatory mediators by binding to the intracellular domain of TLR4 [28]. Because of its suppression of cytokine levels, TAK-242 is known to be a new therapeutic agent for inflammatory diseases [29,30]. Although secreted mucin is expressed constitutively by goblet cells, its production is upregulated by TLR signaling to replenish those degraded by commensals or removed by peristalsis [31]. Our results confirmed the role of TLR signaling in maintaining intestinal barrier function.

An increasing number of studies have identified intestinal dysbiosis–associated gut barrier disruption to be a contributor to renal injury, shedding light on how the gut microbiota drives the pathogenesis of renal disorders [4,32–35]. Antibiotics are among the main causes of acute kidney injury (AKI) worldwide [36]. We expected no damage to the kidney after short-term antibiotic treatment. Of note, after ceftriaxone intervention for just one week, the rats in the Cef group showed higher levels of urinary protein when compared to the CON group. The kidney ultrastructures were further examined by light microscopy and electron microscopy. Under a light microscope, the glomeruli and renal tubules of rats in the three groups were found to be normal; However, electron microscopy revealed that the rats in the antibiotic groups showed obvious podocyte damage. The kidney injury was consistent with the severity of intestinal changes, indicating that intestinal mucosal barrier dysfunction plays a crucial role in the pathogenesis of kidney injury, and TLR signaling may be involved in kidney injury.

## Conclusion

Our data showed dynamic changes in the intestinal microbiota after short-term use of ceftriaxone based on high-throughput sequencing, and revealed the ultrastructure change of both the colon and kidney based on TEM. Our study indicated that short-term antibiotics use may have been underreported, antibiotics played an important role in the gut-kidney axis, by negatively regulating the TLR signaling could have potently beneficial effects in reducing the intestinal mucosal barrier dysfunction and renal injury. Overall, our findings shed some light on the mechanisms underlying intestinal and kidney damage after antibiotic use.

## Data Availability

The datasets used and/or analyzed during the current study available from the corresponding author on reasonable request.
